# Verification of the Effectiveness of a Communication Application in Improving Social Connectedness and Physical Health among Unacquainted Older Men: A Mixed-Methods Pilot Study

**DOI:** 10.3390/ijerph20031884

**Published:** 2023-01-19

**Authors:** Sakino Shinokawa, Hiroki Abe, Risa Takashima, Ryuta Onishi, Michiyo Hirano

**Affiliations:** 1 Graduate School of Health Sciences, Hokkaido University, N12W5, Sapporo 060-0812, Japan; 2 Health & Welfare Department, Higashi Ward Office, N11E7, Sapporo 065-8612, Japan; 3 Faculty of Health Sciences, Hokkaido University, N12W5, Sapporo 060-0812, Japan; 4 Faculty of Nursing, Toyama Prefectural University, 2-2-78, Toyama 930-0975, Japan

**Keywords:** aged, men’s health, information technology, smartphone, communication, exercise

## Abstract

This study aimed to verify the effectiveness of an application (app) in establishing social connectedness among unacquainted older men, as well as improving their physical health. The nine participants were men aged 65 and older in the subarctic zone of Hokkaido, Japan. A mix of quantitative and qualitative methods were adopted as the study design. A questionnaire survey was completed before and after the intervention, and a semi-structured interview was conducted after the intervention. An app-installed smartphone was loaned to the participants, which allowed them to share pictures, voice recordings, and their step count. Quantitative data were analyzed using descriptive statistics and the Wilcoxon signed-rank test, and qualitative data were analyzed using qualitative descriptive analysis to generate categories. The average age of the participants was 77.7 years. The relationship between participants who were interacting for the first time through the app advanced as their understanding of each other’s personalities deepened. The average step count during the third and fourth months was significantly higher than in the first two months. By using the app, older men were able to build relationships with one another. In addition, visualizing the number of steps on the app was effective in improving the number of steps.

## 1. Introduction

Research shows that older adults have fewer social contacts and often feel lonely [1]. Loneliness has been found to be associated with poor physical and mental health [2]; it refers to “the painful subjective feeling—or ‘social pain’—that results from a discrepancy between desired and actual social connections” (p. 2) [3]. Focusing on social connections, O’Rourke et al. stated that “social connections act on caring about others and feeling cared about by others, and feeling of belonging to a group or community” [4]. Interventions and strategies that affect loneliness/social connectedness include engaging in purposeful activity and maintaining contact with one’s social network [5]. In addition, the beneficial effects of physical activity on loneliness are strengthened by social connectedness [6]. Participation in various activities, interaction with others, and a sense of belonging to a group or community, that is, social connectedness, are predicted to be associated with reduced loneliness. Particularly in older men, the personality traits agreeableness, emotional stability, and openness to experiences are negatively associated with loneliness [7]. Therefore, it would be useful to focus on the social connectedness of older adults, especially among men, and to design interventions that provide opportunities for older men to connect with others.

In recent years, information and communication technology (ICT) has been attracting attention as an intervention and a means to help older adults build and maintain connections with others. Tasai et al. developed an application (app) aimed at enabling older adults to use social networking sites to increase social connections, maintain the strength of social connections, and enhance social experiences [8]. It has also been reported that there is a positive association between internet use and mental health among older adults, with enhanced interpersonal interaction at the individual level and increased access to resources within the community [9]. Using ICT is thought to improve social connectedness and mental health in older adults. However, it has been noted that certain forms of online social engagement are linked to higher levels of anxiety and depression [10]. Therefore, it would be important to verify the socially effective use of ICT by older adults in future research.

Regarding ICT use and social connectedness, using ICT reduces feelings of loneliness in older adults [11,12] and makes them feel more connected with friends and family [11]. In a 1-year intervention study, the ICT user group showed significantly decreased loneliness and increased perceived social support and well-being after 6 months, but after 12 months, there was no significant difference between the ICT user and non-user groups [13]. ICT use strengthens social connectedness and reduces feelings of loneliness in older people, but the effects of long-term use have not been detailed. In addition, previous studies have focused on strengthening the existing relationships among older people using ICT [11]. Overcoming loneliness and loss of social connectedness requires acquiring new friends and roles [14]. Therefore, studies should focus on the usefulness of ICT for building social connectedness among unacquainted older adults.

ICT use has positive effects on subjective health, activities of daily living, and daily instrumental activities in older people [15]. A previous study has found that older adults participated in significantly more exercise sessions when they used an exercise app at home [16]. Online group-exercising is effective in motivating older adults [17] and a walking program with online peer support and an app increases physical activity and grip strength in older adults [18]. In addition, the use of ICT by sports directors to provide support increases physical activity in older adults [18,19]. However, older people who are not working use less ICT [20] and are reluctant to use various digital services [21]. Johansson-Pajala et al. suggested the importance of offering ICT solutions with few technical issues, and to provide easily accessible and appropriate support to older adults [12]. Therefore, it is important to consider the usability of ICT among the older population.

Coronavirus disease 2019 (COVID-19) became a global health issue in 2020 and has not been eradicated as of 2023. In a study on ICT use during the pandemic, non-ICT users aged 80 or above and with high loneliness or social isolation scores were more likely to experience cognitive decline [22]. During the COVID-19 pandemic, there is a need to establish strategies to maintain social contact among older adults at high risk of death from infection [23,24,25]. We believe that the ICT used by older adults must target both the improvement of physical health and the maintenance of social connectedness.

Efforts to maintain social connectedness in older adults and reduce their loneliness are important, and we believe that interventions using ICT are effective. Previously mentioned findings revealed that the use of ICT enhances interpersonal interaction, maintains and increases social connectedness, and reduces feelings of loneliness. However, studies assessing the impact of ICT use on social connectedness have often focused on existing relationships, and, to the best of our knowledge, no longitudinal studies to date have explored social connectedness among unacquainted older men. In addition, previous studies have not discussed changes in physical activity in older men, due to the use of ICT without sports directors. The effectiveness of ICT as a means to overcome the loss of social connectedness and deterioration of physical health experienced by older men should be examined. The purpose of this study was to verify the effectiveness of an app in establishing social connectedness among unacquainted older men and improving their physical health. We believe that this research can suggest the possibility of acquiring social and physical health while lowering the risk of infection through communication using an original app between older men who have never met each other.

## 2. Materials and Methods

### 2.1. Design

We applied a combination of quantitative and qualitative methods as a convergent design that involved pre- and post-test comparisons of individuals in a single intervention group. The qualitative descriptive design was used to summarize comprehensive events in daily life [26]. Finally, a joint display integrating quantitative and qualitative data was created and interpreted.

### 2.2. Definition of Terms

O’Rourke and Sidani [4] described social connectedness among older people as “the opposite of loneliness, a subjective evaluation of the extent to which one has meaningful, close, and constructive relationships with others (individuals, groups, society)” (p. 45). Based on this definition, we defined social connectedness as “a recognition that loneliness is either not experienced at all or is experienced less often and a lasting relationship is formed with other members of the group.”

In addition, in this study, the term “older people” has been used to refer to those aged 65 and over.

### 2.3. Research Participants

Participants were recruited through a newspaper, and health and cooking classes. Participants were men over 65 years of age who were not receiving outpatient treatment for dementia and were able to come to the venue by themselves. There were two applicants from the newspaper, and both declined to participate in the research because its content did not meet their needs and they were adhering to COVID-19 precautions. Of the fourteen participants in the health and cooking classes, three declined to participate because their schedule did not align with the study’s timeline, and one opted out owing to their poor physical health during participation. Additionally, one person did not use the app at all; therefore, they were excluded from the analysis target. The final number of research participants was nine. The research area, Hokkaido, has a population of approximately five million, of which approximately 27% are older adults. 

### 2.4. Intervention

**Figure 1:**
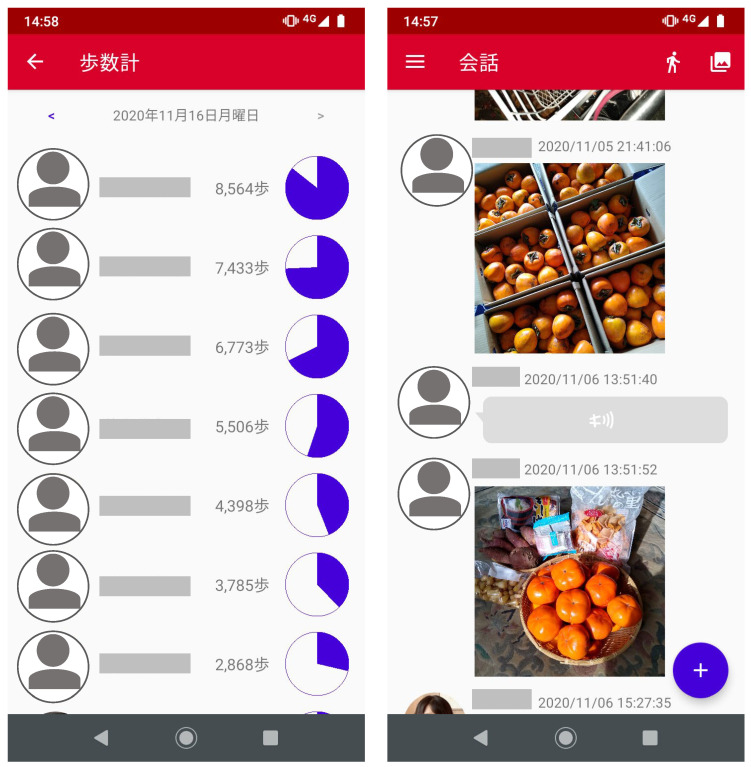
Screenshot of the communication application used in this study. Participants send photos to each other and rank their step count. Note. “歩数計“on the app’s screen represents the step count, “会話“ represents conversation, and "2020年11月16日月曜日" represents Monday 16 November 2020.

A smartphone was shared with the participants for six months, from 16 October 2019 to 15 April 2020. The phone included an installed app called “Kikoeru” [27], designed and developed for older adults in collaboration with an organization where some of the authors work. The app allowed participants to share photos, calculate and share their step count (via a pedometer), and interact with the other participants through a voice-recording feature without having to type. Figure 1 shows screenshots from this app, where members can be seen sending photos to each other and ranking their step count. Each individual’s icon also contained their photo, which allowed other members to see who posted and their steps. Before beginning the intervention, we conducted individual 30 min sessions with each participant to explain how to use the app, distribute instruction manuals, encourage them to use the app freely, and disseminate researchers’ telephone numbers to ensure all participants understood the features. The participants gathered in person once a month in addition to interacting online. However, owing to the COVID-19 pandemic, regular meetings were abandoned, and all interactions were conducted online for two out of the six-month research period.

### 2.5. Data Collection Method

Feelings of loneliness and the step count were used as objective measures of social connectedness and physical health, respectively [28], whereas the subjective evaluation involved measuring participants’ subjective feelings regarding their health. The intervention lasted for six months. Participants completed a self-administered questionnaire survey on subjective health and loneliness before (October 2019) and after the intervention (between April and July 2020), which was followed by semi-structured interviews.

#### 2.5.1. Self-Administered Questionnaire Survey

The questionnaire items collected information regarding basic participant attributes, subjective feelings related to health, and experiences of loneliness. The basic attributes included age, living arrangement, years spent in the current residence, educational qualifications, worries about personal finances, and the level of care needed. The level of care needed was based on the long-term care insurance system in Japan, which divides the need for care into seven stages: individuals in the first two support levels require limited help, while those in the other five require extensive help. The higher the level, the greater the assistance needed.

The participants’ subjective feelings regarding their health were measured using the following item: “On a scale of zero to ten, where zero means ‘unhealthy’ and ten means ‘very healthy,’ how would you rate your own health condition?” The higher the score, the healthier the participants consider themselves to be.

Feelings of loneliness were measured using the Ando, Osada, and Kodama (AOK) Loneliness Scale [29] consisting of 10 items, with scores ranging from zero to ten. A score of zero indicated no feelings of loneliness, whereas a score of one or more indicated the presence of feelings of loneliness. The higher the score, the stronger the participants’ feelings of loneliness. Although the scale’s reliability for the current study could not be established meaningfully, owing to the small sample size, the Cronbach’s α of this scale was 0.89 in a previous study on older people, thus confirming its reliability [30].

#### 2.5.2. Semi-Structured Interview

The interview was conducted in a private room of the district hall or at the participant’s home according to convenience, and was recorded on a digital voice recorder after obtaining consent. All interviews were conducted by the first author. The participants were asked, “Did you ever feel connected to other members after using the app?” In response to the question, in order to further deepen the participants’ narratives, we asked them, “What kind of connection did you feel?” and “When did you feel the connection?” Participants were encouraged to openly share their experiences with their connections. The average interview time was 52 min.

#### 2.5.3. Logs from the Server

Logs regarding the participants’ step count and the number of times the app was accessed were collected from the application server.

### 2.6. Data Analysis

#### 2.6.1. Self-Administered Questionnaire Survey

Descriptive statistics showing changes were used to analyze questionnaires before and after the intervention. We calculated the average score to show the changes in results. The IBM SPSS Statistics 25.0 Exact test (IBM, Armonk, NY, USA) was used as analysis software to handle the small sample size, and the Monte Carlo simulation was used to estimate significance. We used Wilcoxon’s signed-rank test to identify changes in subjective perceptions of health and loneliness.

#### 2.6.2. Semi-Structured Interview

The qualitative descriptive study was used to summarize comprehensive events in daily life [26]. The recorded interviews were transcribed and coded to examine the social connectedness built by participants through the app. Next, while focusing on the context of the data, similar codes were aggregated to create subcategories, and the similarities and differences between the subcategories were examined and integrated to extract the final categories. All analyses were performed manually by the first author. The extracted codes, subcategories, and categories were analyzed iteratively until the first and last authors agreed. The first author is a graduate student in a master’s program with a nursing qualification, and the last author is a qualified nurse and public health nurse with experience in qualitative research. The results of the analysis agreed upon by the first and last authors were shared by the other authors, and those agreed upon by all authors were used as subcategories and categories. The categories were then checked for authenticity by the participants.

#### 2.6.3. Logs from the Server

The average step count taken every two months during the intervention period was calculated, and the Monte Carlo simulation was used to estimate significance. Monte Carlo calculates the likelihood of a given range of outcomes occurring. Therefore, in this study, we used Monte Carlo to calculate the probability of an increase or decrease in the number of steps during a given period. During the fifth and sixth months of the intervention (15 February 2020 to 16 April 2020), the period of behavioral restrictions, such as refraining from going out, related to COVID-19 in Hokkaido (28 February 2020), was duplicated. Steps taken in these months were excluded because they were affected by the request.

#### 2.6.4. Combined Results from Quantitative and Qualitative Methods

Mixed-methods research is a type of research in which the investigator collects and analyzes data, integrates the findings, and draws inferences using both qualitative and quantitative approaches or methods in a single study or a program of inquiry [31]. Referencing mixed-methods research, this study integrated qualitative and quantitative data to create a joint display to verify the effectiveness of an app in establishing social connectedness among unacquainted older men, as well as improving their physical health, and to examine the results of the intervention. 

### 2.7. Ethical Approval and Consent to Participate

In this study, participation was voluntary. Participants were informed that the data obtained would only be used for the study, and their confidentiality and anonymity were guaranteed. Written consent was obtained from all participants. This study was approved by the Ethics Review Committee of the Faculty of Health Sciences, Hokkaido University (1 July 2019, approval number 19-36).

## 3. Results

### 3.1. Characteristics of Research Participants

Table 1 provides an overview of the participants’ attributes. Participants’ age range was 70–84 years, with an average age of 77.67 ± 4.14 years.

### 3.2. Usage of the Application

Table 2 shows the number of days the app was used. After the fourth month of the intervention, eight of the nine participants used the app 80% of the days during the period. The average number of posts per month was 95.33, and the average number of views of posts was 1959.83.

### 3.3. Social Connectedness Established through the Use of the App

Table 3 shows categories and subthemes representing social connectedness through the app. We extracted sixty codes, seventeen subcategories, and six categories from the social connectedness built using the app.

#### 3.3.1. Category 1: Sharing Posts Deepens Understanding and Impressions

Participants often submitted photos of flowers and landscapes as well as their voice recordings with descriptions of them. By sharing their photos and voices, members experienced perspectives that they could not perceive on their own. Additionally, the sounds of daily life emitted from the posts presented an opportunity to learn about the members’ lives. Moreover, participants learned about other members’ personalities and activities, deepening their interest in the members’ lives.

*“Participant I often participates in various events and posts… It seems that he has a sore throat, but he often takes various pictures and posts them, so I understand the pictures easily, even without his voice. It is easy to understand what he is doing.”—Participant A*

#### 3.3.2. Category 2: Familiarity Advances the Relationship

Participants obtained a sense of the other members’ lifestyles from the content of their postings, and became acquainted with the other members’ personalities. Therefore, the members felt comfortable talking with each other when they met in person. By listening to members’ posts on the app and learning about their daily lives, participants became familiar with the members and wanted to become better acquainted. Participants felt that they wanted to cherish “this encounter” that they had taken the time to create together.

*“I want to cherish the long-awaited encounter because we met and worked together by some kind of fate.”—Participant* *F*

#### 3.3.3. Category 3: Being Interested in the Unfiltered Lives of the Members

Participants gained a better understanding of who the members were by sharing posts. Therefore, participants mentioned wanting to not only see the posts that looked good but also discover the person’s true nature because they could understand the members through their posts.

*“The number of steps participant H takes daily is amazing. How does do it?”—Participant B*

#### 3.3.4. Category 4: Wanting Other Members to Gain Interest

Participants preferred to post about special activities, such as rare photos taken at travel destinations to gain attention from other members, and did not share many of their daily events. Many participants posted respectable activities and photos, and participants who did not perform such activities were hesitant to post. They wanted to post something that would inspire other members rather than their own daily lives.

*“When members provide a topic such as a flower series or a cooking series, I want to get on it, but I have not done any cooking to take a photo... I know that daily meals are fine, but there is just something about posting them that makes me feel self-conscious.”—Participant B*

*“I go to play table tennis as soon as the gymnasium opens at 8:30 am. I wish I could take a picture that would make everyone thinks “How can an older person like them (participant H) do this?””—Participant H*

#### 3.3.5. Category 5: Feeling Motivated to Stay Active

On the app, participants shared good activities such as their daily steps, exercise classes they attended, and volunteer activities. Participants felt that the members were doing their best and were inspired to improve their own daily routines.

*“They are doing what I aim for. If I think they’re doing great at something, I want to make an effort to get as close as possible to it.”—Participant H*

*“By looking at the app, I can see that Participant H is walking more. I also want to increase the number of steps I take, so I take more walks than in the past. When visiting an acquaintance, I often walk instead of driving.”—Participant I*

#### 3.3.6. Category 6: Wanting More Than the Level of Interaction through the App Alone

During the COVID-19 pandemic, when the participants were unable to meet, they felt a sense of closeness through the app posts, but also frustration at not being able to meet. As only good activities were shared through the app, rather than members’ daily lives, participants wanted to meet and interact in person. Sharing posts on the app helped them create topics of conversation when they met in person. Thus, interacting through the app and meeting in person were complementary.

*“For now (because I cannot meet the members owing to COVID-19), I can only get in touch with them through this app, so I am still a little dissatisfied.”—Participant C*

### 3.4. Loneliness

The results of the AOK Loneliness Scale, and the pre- and post-intervention comparisons of the scores are presented in Table 4. Although no significant difference was found after the intervention, the average loneliness scores before and after the intervention were 1.00 ± 1.25 points and 0.77 ± 0.79 points, respectively.

### 3.5. Impact of ICT Use on Health

#### 3.5.1. Step Count

Table 5 shows the change in participants’ step count. The average step count in the third and fourth months was significantly higher than that of the first two months (5840.22 ± 4840.54 versus 4780.78 ± 3977.66; *p* = 0.038).

#### 3.5.2. Subjective Health

Participants’ ratings of their subjective health are shown in Table 4. Although no significant difference was found after the intervention, the average subjective health ratings before and after the intervention were 7.1 ± 2.18 points and 7.0 ± 1.33, respectively.

#### 3.5.3. Combined Results from Quantitative and Qualitative Methods

Table 6 shows the integration of qualitative and quantitative data and the social connectedness that has been established by participants to improve their physical health.

## 4. Discussion

Our results suggest that older men are able to establish new connections and increase their step count over a period of six months by using an app designed specifically for them. These results are discussed in further detail below.

### 4.1. New Social Connectedness Established through the App

Previous studies have indicated that older adults seek opportunities to share their feelings online with peers who have had similar experiences, such as retirement and the loss of interpersonal relationships [32]. The interaction through the app seemed to satisfy this need among participants in the present study.

Furthermore, participants wanted other members to show interest in them; consequently, they chose to post about the positive aspects of their lives. They became gradually interested in understanding other members and deepening their future friendships. Studies have reported that ICT is helpful in maintaining offline social relationships in addition to online ones [33]. According to Vošner et al. [32], men want to see who they are talking to and tend to value face-to-face interactions more than women do. Moreover, online interaction tends to be superficial [34]. This is because, in comparison to women, men find it more challenging to express their feelings to their friends and often remain superficial, making it difficult for them to reveal their true nature [35]. Because many of the participants did not post on a daily basis, it is assumed that face-to-face interaction was also required to understand the characters of the members. There were no potential or overt adverse effects of app connections in this study.

Furthermore, no increase was observed in the participants’ score on the Loneliness Scale. In a previous study, the average loneliness score for men was found to be 1.74 ± 2.44 points [29]. However, participants in this study experienced a lower level of loneliness even before the intervention. This low level of loneliness may be related to the fact that all participants had cohabitants, as having a cohabitant was associated with lower levels of loneliness in a previous study [36]. In another survey conducted in Japan in April 2020, when the post-intervention questionnaire was completed, more than 40% of the adults reported feeling lonely owing to a loss of contact as a result of the COVID-19 restrictions [37]. However, most participants in this study were able to maintain the status quo and did not experience increased feelings of loneliness, even in such extraneous circumstances. Contrastingly, participants A and C felt lonelier after the intervention. Participant A said that he felt lonely when no one responded to his post and wanted more replies from the members. This may have been a result of participant A feeling uneasy about the content of his post, which was not masculine [38], when there was no response from the members. Participant C seemed to feel lonely because he could not meet the members, directly owing to COVID-19 restrictions, as shown in Category 6. It is thought that the decrease in the frequency of interpersonal contact affected loneliness [35] owing to movement restrictions during the COVID-19 pandemic.

As found in previous studies, men hesitate to express their emotions [39]. Compared with women, men rarely form emotional connections, and when connections are formed, they tend to be shallow [38]. Thus, the app is useful as an auxiliary tool to promote mutual understanding among unacquainted members and facilitate the development of sustainable relationships among them.

### 4.2. Impact of ICT Use on the Health of Older Men

Participants showed a significant increase in their step count during the third and fourth months of the intervention compared with the first two months. The target area is located in the subarctic zone. The third and fourth months of the intervention occurred during the time of year when older adults’ activities generally decreased due to snow and cold [40]. However, the participants in our study consciously walked more and increased their step count.

One reason for the increase in participants’ step count could be the presence of a goal. Several participants said in interviews that they set their own goals, even though the researchers did not ask them to do so. Studies have shown that the use of a pedometer facilitates numerical goal setting and can increase the step count with little conscious effort [41]. As the step count is directly linked to the degree of daily activity and is displayed numerically, it is easy for older people to evaluate their level of achievement against the goal [42]. Furthermore, the sense of accomplishment experienced upon reaching the targeted step count could help maintain motivation to be consistent. In addition, the study found a significant difference in the mean number of steps after the intervention but no significant difference in the subjective health scores of the participants. A significant positive relationship between ICT use and subjective health status has been reported [37]. However, in this study, the participants’ subjective health evaluation was high even before the intervention started, and it was different from the characteristics of participants in the previous study; therefore, we think that a significant relationship could not be found.

Interactions with ICT provide many opportunities for older adults to observe and imitate the online behavior of other members [43]. Participants who saw other people’s posts were motivated to improve their physical activity. From this, it was possible to increase the motivation for physical activity and increase the number of steps through interaction with people of the same generation. By establishing connections, participants could recognize others by their higher step count, and try to improve their own step count accordingly. This psychological state is often referred to as “affiliation motivation,” and Leary and Hoyle [44] described it as a desire to associate and interact with other people, particularly in warm, harmonious ways. Therefore, we believe that affiliation motivation worked to maintain participants’ relationships (i.e., to engage in the same actions). Additionally, affiliation motivation contributes to health promotion [45]. The connections established by using ICT-induced affiliation motivation contributed to the improvement in participants’ step counts and the maintenance of their subjective health.

### 4.3. Limitations

The study had certain limitations. First, owing to the small sample size, the quantitative data gathered were limited. It is necessary to secure a larger sample size and include a control group that does not use ICT in future research. Second, the participants in this study experienced lower levels of loneliness and better subjective health even before the intervention. In addition, as they were recruited through newspapers and health and cooking classes, they were prior participants in cooking and health classes. In the evaluation of the target population, the connections made using the app may be different for those who engage in little to no social activities. In future research, further verification of participants will be required to include those who experience greater loneliness, have poor subjective health, and partake in less social activities. 

## 5. Conclusions

In this pilot study, the app provided an opportunity for participants, who interacted with each other for the first time using the app, to build social connectedness. It led to mutual understanding and helped them build favorable relationships with each other at an early stage. Furthermore, it led to an increase in their step count by allowing them to share their experiences and activities, thus promoting healthy behaviors among them. The results of this study suggest that older men may be able to effectively use apps developed for the elderly to recognize new connections, alleviate worsening loneliness, and induce more physical activity.

## Figures and Tables

**Table 1:** Participants’ overview.

ID	Age	Living Arrangements	Duration of Residence (Years)	Care Need Levels	Final Education	Economic Situation	Previous Smartphone Experience
A	70s	with spouse, child, and other family members	over 10 less than 20	not applicable	junior college or university	slightly worried	Yes
B	70s	with spouse, child, and other family members	over 20 less than 30	not applicable	university	slightly worried	Yes
C	70s	with spouse	over 20 less than 30	not applicable	high school	not too worried	Yes
D	70s	with spouse	over 20 less than 30	not applicable	junior college or university	not too worried	No
E	70s	with spouse	over 30 less than 40	support level 2	junior high school	not too worried	Yes
F	80s	with spouse, child, and other family members	over 10 less than 20	not applicable	junior high school	slightly worried	No
G	80s	with spouse	over 30 less than 40	not applicable	junior college or university	not too worried	No
H	80s	with spouse	less than 10	not applicable	high school	not too worried	No
I	80s	with spouse	over 30 less than 40	support level 1	junior high school	not too worried	No

**Table 2:** Number of days the participants used the communication application (app).

	1st Month	2nd Month	3rd Month	4th Month	5th Month	6th Month
ID	(Number of Days: 31)	(Number of Days: 30)	(Number of Days: 31)	(Number of Days: 31)	(Number of Days: 29)	(Number of Days: 31)
A	31	30	31	31	29	31
B	31	30	31	31	29	31
C	30	29	29	28	23	28
D	27	5	11	15	11	15
E	24	30	28	25	28	30
F	30	25	29	28	27	26
G	19	28	29	31	29	31
H	25	30	30	31	29	31
I	2	21	21	31	29	31

**Table 3:** Themes and subthemes representing social connectedness established through the app.

Category	Subcategory	Representative Quote
Sharing Posts Deepens Understanding and Impressions	Development of greater respect for others	Participant I frequently attends various events and posts about them… It seems that he has a sore throat, but he often takes various pictures and posts them, so I understand the pictures easily, even without his voice. It is easy to understand what he is doing. (Participant A)
	Listening to each other’s posts deepened their understanding of the members	I am inspired by other people’s posts. Even if it is the same scenery or photo, I feel that there are different ways of feeling and seeing. (Participant F)
	Self-projecting onto other members’ posts and finding them relatable	I think a post like that (where we can hear their wife’s voice in the background) is fine. I think it’s great that it conveys a sense of life. (Participant C)
Familiarity Advances the Relationship	Familiarity with the members, by way of knowing what they are doing, later led to deeper face-to-face conversations	I can see that other people are absent on Saturdays and Sundays, and on the contrary, there are people who are active on Saturdays and Sundays. I wonder what kind of person he is. It may be easier to meet and talk because I understand that. (Participant I)
	Interaction through the app laid the foundation for face-to-face conversation	The other day, I happened to sit next to one of the members at a community gathering New Year’s party. It was then that he realized for the first time that she belonged to the same group. (Participant G)
	The desire to become acquainted with each other grew during interactions through the app	I want to cherish the long-awaited meeting because we met and worked together by chance. (Participant F)
	Sharing posts led to the desire to keep the group active	After listening to the post, I thought that I would like to cooperate with everyone who has the same idea to liven up the meeting. (Participant G)
	Feeling a sense of familiarity through online interaction, even without meeting	Even if I cannot meet or go out because of the new coronavirus infection, it is fun to connect with the members by using the communication app. (Participant E)
Being Interested in the Unfiltered Lives of the Members	Wanting to know the voice of the members, not just see posts that look good	I think we can share our feelings. (I think it’s better to post what I think, even if I stumble or use short sentence.) (Participant C)
	Thinking about the members after seeing the posts and number of steps	The number of steps participant H takes every day is amazing. How does he do it? (Participant B)
Wanting Other Members to Gain Interest	Wanting to share things that look good, not just daily life activities	I can take unusual pictures if I go to a strange place. Because I cannot go out owing to COVID-19, I do not have any pictures to post. (Participant C)
	Feeling pressured to make a proper post	When members provide a topic, such as a flower series or a cooking series, I want to get on it, but I have not done any cooking to take a photo... I know that daily meals are fine, but there is just something about posting them that makes me feel self-conscious. (Participant B)
	Wanting to positively impact other members through the app	I go to play table tennis as soon as the gymnasium opens at 8:30 am. I wish I could take a picture that would make everyone thinks “How can an older person like them (participant H) do this?” (Participant H)
	Feeling glad about other members commenting on the posts	I’m glad that everyone praised my step count. (Participant H) I feel lonely when I don’t get a reaction to my post. (Participant A)
Feeling Motivated to Stay Active	Wanting to challenge each other with the number of steps walked and other posts, and make changes in their own life	They are doing what I aim for. If I think they’re doing great at something, I want to make an effort to get as close as possible to it. (Participant H)
	Wanting to get a reaction to their own daily activities and being able to sympathize with members	I want to publicize what I have done so far, such as what kind of conversation is necessary when interacting with people, how I have maintained my health, etc. (Participant G)
Wanting More Than the Level of Interaction Through the App Alone	The relationship cannot be developed by online interaction alone	For now, because I cannot meet the members owing to COVID-19, I can only get in touch with them through this app, so I am still a little dissatisfied. (Participant C)

**Table 4:** Participants’ loneliness scores and subjective health status.

	Loneliness		Subjective Health Status	
ID	Before Intervention	After Intervention	Before Intervention	After Intervention
A	0	2	3	6
B	0	0	8	7
C	1	2	9	9
D	1	1	8	8
E	4	0	5	4
F	2	1	7	7
G	1	1	9	7
H	0	0	10	7
I	0	0	5	8

Note. Loneliness was measured using the Ando, Osada, and Kodama (AOK) Loneliness Scale, score range 0–10 (0 = no feelings of loneliness; 1 ≤ feelings of loneliness present). The subjective health status score range was 0–10 (0 = unhealthy; 10 = very healthy).

**Table 5:** Change in participants’ step count.

ID	1st Month	2nd Month	3rd Month	4th Month	
A	3412.45	4185.23	3888.81	4460.45	
B	9809.76	10432.9	11180.61	12021.52	
C	3005.77	2102.43	1651.16	1742.74	
D	5235.32	4432.44	5112.52	5940.26	
E	3474.96	6071.9	5098.81	5289.11	
F	3417.48	3215.7	3760.29	2873.72	
G	580.87	2354.63	2548.87	1775.45	
H	9812.9	14323.5	14641.23	17579.84	
I	0.18	545.57	275.35	538.26	
	1st and 2nd month		3rd and 4th month		*p* value
	(Mean ± SD)		(Mean ± SD)		
Number of steps	4780.78 ± 3797.66		5840.22 ± 4840.54		0.038 ^a^

Note. ^a^: Calculated using the Wilcoxon signed rank test.

**Table 6:** Integrated quantitative and qualitative results.

Goal	Quantitative Results	Qualitative Results	Interpretation
Establishing social connectedness	The average loneliness scores before and after the intervention were 1.00 ± 1.25 points and 0.77 ± 0.79 points, respectively.	Category 1: Sharing Posts Deepens Understanding and Impressions Category 2: Familiarity Advances the Relationship Category 3: Being Interested in the Unfiltered Lives of the Members Category 4: Wanting Other Members to Gain Interest Category 6: Wanting More Than the Level of Interaction Through the App Alone	At the time of the baseline assessment, participants had a low level of loneliness. As all the participants lived with their families, it is probable that a low level of loneliness was maintained. However, during the intervention period, the COVID-19 infection spread, which included the period of quarantine. Therefore, the feeling of loneliness was likely to increase during the period of high COVID-19 infections. However, it is presumed that participants were able to maintain a low level of loneliness even during the COVID-19 pandemic by building social connectedness with the members. Unlike social isolation, loneliness is a subjective index; therefore, interacting with members through the app could enable them to feel a connection with people. As shown in Category 6, it can be difficult to maintain favorable connections among participants with long-term ICT-only interactions.
Improving their physical health	The average number of steps in the third and fourth months was significantly higher than that of the first two months (5840.22 ± 4840.54 versus 4780.78 ± 3977.66, respectively; *p* = 0.038). The average subjective health rating before the intervention was 7.1 ± 2.18 points and that after the intervention was 7.0 ± 1.33 points.	Category 4: Wanting Other Members to Gain Interest Category 5: Feeling Motivated to Stay Active	Participants wanted to post and have a positive health impact on other participants who viewed it. Participants who saw the posts of others found an opportunity to improve their physical activities. Furthermore, it is possible that the influence of the affiliation motivation to approach the state and maintain the relationship by being aware of others who have a higher step count contributed to their own increases.

## Data Availability

The data presented in this study are available on request from the corresponding author. The data are not publicly available, because consent for release of the data has not been obtained from the participants.
